# Tick Intrastadial Feeding and Its Role on IgE Production in the Murine Model of Alpha-gal Syndrome: The Tick “Transmission” Hypothesis

**DOI:** 10.3389/fimmu.2022.844262

**Published:** 2022-03-04

**Authors:** L. Paulina Maldonado-Ruiz, Gunavanthi D. Boorgula, Donghun Kim, Sherry D. Fleming, Yoonseong Park

**Affiliations:** ^1^ Department of Entomology, Kansas State University, Manhattan, KS, United States; ^2^ Department of Entomology, Kyungpook National University, Daegu, South Korea; ^3^ Division of Biology, Kansas State University, Manhattan, KS, United States

**Keywords:** *Amblyomma americanum*, ticks, alpha-gal, red meat allergy, alpha-gal syndrome

## Abstract

Recent studies have provided strong evidence indicating that lone star tick bites are a cause of AGS (alpha-gal syndrome, also known as red meat allergy RMA) in humans. AGS is characterized by an increase in IgE antibody production against galactose-alpha-1,3-galactose (aGal), which is a common glycan found in mammalian tissue, except in Old World monkeys and humans. The main causative factor of AGS, the lone star tick (*Amblyomma americanum*), is broadly distributed throughout the east and midwest of the United States and is a vector of a wide range of human and animal pathogens. Our earlier glycomics study of the salivary glands of partially fed male and female ticks revealed relatively high levels of aGal epitopes. In this study, we found that partially fed males of *A. americanum* on bovine blood, which engage in multiple intrastadial feedings, carry a large amount of aGal in the salivary glands. In our current work, we aimed to test whether ticks mediate the transmission of the aGal sensitizer acquired from nonhuman blood to humans in the intrastadial host switch (referred to as the “transmission” hypothesis). To test this hypothesis, we used an alpha-galactosyltransferase knockout mutant mouse (aGT-KO) model system infested with ticks that were unfed or partially fed on bovine blood. Based on the levels of total IgE and specific IgG and IgE antibodies against aGal after tick feedings, aGT-KO mice significantly responded to tick feeding and injection of aGal (Galα1-3Galβ1-4GlcNAc) conjugated to human serum albumin or mouse serum albumin (aGal-HSA or aGal-MSA) by increasing total IgE and aGal-specific IgE levels compared to those in C57BL/6 control mice. All of the treatments of aGT-KO mice involving the feeding of partially fed and unfed ticks functioned as sensitizers that increased the levels of specific IgE against aGal, with large individual variations. The data in this study do not support the “transmission” component of AGS, although they confirmed that aGT-KO mice can be used as a model for RMA studies.

## Introduction

In humans, alpha-gal syndrome (AGS and also known as red meat allergy RMA) is caused by the increased production of IgE antibodies against galactose-alpha-1,3-galactose (alpha-gal or aGal) (1). This glycan is found in most mammals, with the exception of Old World monkeys and humans, since the alpha-1,3-galactosyltransferase gene (α1,3GT) has been inactivated in ancestral Old World primates in the course of evolution (2, 3). AGS or RMA is observed in susceptible individuals after the ingestion of red meat, with a clinical manifestation of urticaria and anaphylaxis (4). Worldwide reports have found that aGal allergic responses are directly linked to an initial sensitization by tick bites of different species: *lxodes ricinus* in Europe, *l. holocyclus* in Australia, *Hemaphysalis longicornis* in Asia, *Amblyomma americanum* and other *Amblyomma* species in North America and South America, respectively (5–11). In the United States, only lone star tick (*A. americanum*) bites have been identified as the primary causal factor for RMA (12, 13). Cases of RMA have been reported in the southeastern United States, including the regions known for the distributions of the lone star tick (14). Recent studies have shown that the salivary glands of *A. americanum* contain N-glycosylated protein(s) with galactose alpha-1,3-galactose beta-1,4-N-acetylglucosamine (aGal) (11, 14–16). The source of the aGal sensitizer in tick saliva awaits further elucidation of the molecular identity and the signaling pathway leading to aGal sensitization.

The presence of glycosylated proteins carrying aGal in tick saliva might not be the sole determinant of aGal sensitization in AGS. For example, aGal N-glycan was also found in the salivary glands of *I. scapularis* in addition to those of *A. americanum* (17), while there are no supporting data for the bites of *I. scapularis* causing AGS. Likewise, a remaining question is that AGS occurs in only a small subgroup of the overall human population that experiences tick bites, which indicates that either the ticks under specific conditions are culprits of change or subpopulations of humans are vulnerable to the development of IgE against aGal, causing AGS. Therefore, the variations in the presence of the aGal sensitizer in tick salivary secretion and additional unidentified factors need to be further investigated in AGS.

A factor could stem from tick feeding behaviors that lead to a greater chance of feeding on humans, which are not a natural host. The males of *A. americanum* are known to have multiple intrastadial feedings, while female ticks are generally engaged in a single feeding event on a single host for full engorgement (18). We speculated that this behavioral difference of male ticks could provide a greater opportunity for an intrastadial switch in hosts from a nonhuman mammal to a human. Furthermore, we found that the salivary glands of male ticks that fed on bovine blood *via* artificial membrane feeding contained large amounts of aGal epitopes in this study. We employed alpha-galactosyltransferase knockout mutant mice (aGT-KO) to test the differences in the responses to ticks that were partially fed on bovine blood. The results showed that the aGT-KO mouse responded to tick feeding by increasing total IgE, specific IgG and IgE levels, but with large individual variations, without sufficient levels of discrimination for feedings by partially fed ticks.

## Methods

### Mice

The C57BL/6 mouse strain and alpha-gal-knockout (aGT-KO; C57BL/6/CimlKvl-Tgaltm1Tea, obtained from the Scripps Research Institute, Janda Lab, La Jolla, CA) were maintained in the AAALAC-approved animal facility of the Laboratory Animal Care Service (LACS) at Kansas State University. All animal protocols were approved and compliant with the guidelines of the Institutional Animal Care and Use Committee (IACUC). The founders were tested for the genotype by using PCR for the mutation.

The study was divided into two experiments: Experiment 1) was sensitization to alpha-gal by various feeding stages of *A. americanum* tick bites, and Experiment 2) was sensitization to the feedings of different species of ticks. For Experiment 1, we used eighty-two aGT-KO mice (40 females, 72 males) and 48 C57BL/6 wild-type mice (24 females, 24 males). For Experiment 2, we used 58 aGT-KO mice (31 females, 27 males). All mice used were 8-12 weeks old at the time of the first sampling. Mice were anesthetized with isoflurane (2.5-5%) (Akorn Pharmaceuticals, IL, USA) *via* inhalation at each sampling point (blood draw) and euthanized immediately after the final blood collection by cervical dislocation.

### Ticks and Tick Prefeeding

Unfed (naïve) 2- to 3-month-old adult male and female *A. americanum*, *Dermacentor variabilis*, and *Ixodes scapularis* ticks were obtained from the Oklahoma State University tick rearing facility. All ticks were kept at room temperature and >95% RH until use in experiments. For the treatments that required partially fed ticks, the ticks were prefed (4–5 days blood fed) with defibrinated bovine blood (Hemostat Laboratories, CA, USA) using a modified artificial feeding system (19) before being infested onto the mice.

### Western Blotting of the Salivary Glands of *A. americanum* for Detection of aGal Epitope

Ticks were prefed on bovine blood for 4 days in an artificial membrane feeding system and used for dissections of the salivary glands. Three pairs of salivary glands were pooled for isolation of protein for electrophoresis. Unfed male and female salivary glands and dorsums were included, and 10 µg of bovine thyroglobulin was used as a positive control, as it is known to contain alpha-gal epitope (20). Proteins were extracted using the protein extraction reagent T-per™ (Thermo Scientific, MA, USA), and an aliquot (~5-10 µg of total protein) was treated with, PNGase F or alpha-1,3 galactosylase enzymes following the manufacturer’s protocol for digestion. PNGase F (New England Biolabs, MA, USA) was used for deglycosylation of all N-glycans, including the aGal epitope or alpha-1,3 galactosylase (P0731S, New England Biolabs, MA, USA), which exclusively cleaves the terminal aGal. Samples were loaded for SDS-polyacrylamide gel electrophoresis at ~5 μg of protein per lane using a Mini-PROTEAN^®^ TGX Stain-Free™ gel (Bio–Rad, CA, USA), and Precision Plus Protein™ Unstained standard (10–250 kDa) was used as a protein size marker (Bio–Rad). The gel was transferred to a ready to use pre-wetted PVDF membrane (Bio–Rad, USA) using a Trans-Blot^®^ Turbo™ transfer System (BioRad) and blocked with 3% bovine serum albumin in phosphate-buffered saline supplemented with 0.05% Tween (PBST). Immunoblotting was conducted using an IgM mouse monoclonal antibody against the aGal epitope (M86) (Enzo Life Sciences, NY, USA) at 1:10 with overnight incubation. Goat-anti-mouse IgM conjugated to HRP was used as a secondary antibody at 1:500 (Novus Biologicals, CO, USA) for 3 hours and followed by incubation with the substrate 3,3′-Diaminobenzidine (0.05%) with 0.003% hydrogen peroxide in PBST. Gel image was analyzed by using the ImageJ software version 1.8.1_172 (21) for measuring the difference between pixel values. Lane with no immunoreactivity was used for subtraction of background. Thyroglobulin (suggested to contain ~4 molecules of aGal per protein molecule, or ~60 pmoles of aGal in 10µg) (22) was used to calculate an approximation of aGal concentration within the tick salivary glands.

### Effects of Tick Feedings on aGT-KO Mice

#### Experiment 1: aGal Sensitization by *A. americanum* Tick Bites in aGT-KO Mice

**Figure 1:**
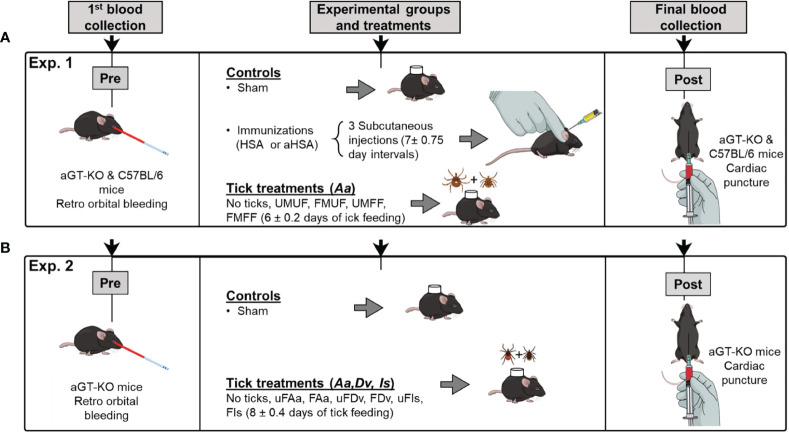
A schematic diagram of the experimental design. **(A)** section outlines the treatments and structure for assessing RMA sensitization using pairs of *Amblyomma americanum* ticks (Experiment 1). The treatments were varied with partially fed and unfed (F,U) male and female (M,F), aGT-KO mice and C57BL/6 control mice. **(B)** experimental design for assessing comparisons between sensitization through bites of other ixodid tick species (Experiment 2), fed (F) and unfed (uF) ticks, *Amblyomma americanum* (Aa), *Dermacentor variabilis* (Dv), and *Ixodes scapularis* (Is). In all treatments involving ticks, male and female pairs of ticks were used.

For the aGT-KO homozygous mice, we included three controls: 1) sham control: mice were shaved on the back and tick feeding chamber was placed without ticks, 2) Human serum albumin (HSA) control: mice were injected with HSA (25 µg, with no alpha-gal), and 3) aGal conjugated HSA (aHSA) control: mice were injected with 25 µg aHSA. Subcutaneous injection was performed 3 times at 7 ± 0.75 day intervals, as previous reports show that weekly immunizations for 3 to 4 weeks, resulted in increased IgE against the aGal sensitizer (8). Four other experimental groups were combinations of male (M) and female (F), partially fed (F) and unfed (U) ticks. Therefore, four treatments were the mice infested with one pair of ticks UMUF, FMUF, UMFF, and FMFF (**Table 1**). The control groups with wild-type C57Bl/6 mice were the sham control and injections with HSA or aHSA and the treatment with partially fed males plus partially fed females (FMFF) (**Table 1**). Mice were prebled by retro orbital bleeding before treatment using a 1.1 mm diameter capillary tube (100-300 µl) to quantify the pretreatment immunoglobulin levels (**Figure 1A**) and by cardiac puncture in the posttreatment using a 1 ml syringe with a 25 gauge needle (~1 ml).

**Table 1:** Experimental groups assigned to mice and tick pre-treatments for experiment 1.

Mice	Group	Pre-treatment of ticks	Abbreviation	Male	Female
aGT-KO	Control 1	No tick feeding only Shave + chamber	Sham	5	5
	Control 2	Human serum albumin immunization	HSA	5	5
	Control 1	Human serum albumin + alpha-gal immunization	aHSA	5	5
	Exp. Group 1	Unfed Male, Unfed Female	UM, UF	8	7
	Exp. Group 2	Fed Male, Unfed Female	FM, UF	7	7
	Exp. Group 3	Unfed Male, Fed Female	UM, FF	6	6
	Exp. Group 4	Fed Male, Fed Female	FM, FF	6	6
C57BL/6	Control 1	No tick feeding only Shave + chamber	Sham	6	6
mice	Control 2	Human serum albumin immunization	HSA	6	6
	Control 3	Human serum albumin + alpha-gal immunization	aHSA	6	6
	Exp. Group 1	Fed Male, Fed Female	FM, FF	6	6

Ticks were placed on mice inside a 15 mm circular chamber made from double layer of double sided mounting tape (Scotch, 3 M, MN, USA) ~4 mm thick covered with nylon mesh. The chamber was glued on the back with 3 M Vetbond™ after shaving the fur and covered with 3 M Tegaderm™ adhesive tape. Finally, the chamber was secured with a mouse jacket (Harvard Apparatus, MA, USA). In the daily monitoring, if the ticks were not attached to mice within a week or died, the ticks were replaced with a new pair until successful tick attachment was observed. Ticks were removed between 4-8 days after attachment (**Figure 1A**) when the feeding ticks reached the rapidly engorging stage to repletion.

#### Experiment 2: Alpha-Gal Sensitization in Mice by Bites of Other Ixodid Tick Species

For Experiment 2, only aGT-KO mice were used, and the experimental groups included only the “sham” control group and three different species of partially fed (F) or unfed (uF) ticks. Three different species were *A. americanum* (Aa), *Dermacentor variabilis* (Dv), and *I. scapularis* (Is), which amounted to six experimental groups (uFAa, FAa, uFDv, FDv, uFIs, and FIs).

### Quantification of Immunoglobulins by ELISA

For total IgE quantification, diluted mouse serum (1:50) was used in an ELISA assay. For the specific IgE quantification assay, IgG was depleted from mouse sera by using protein G resin (Genscript, NJ, USA) at a 1:1 ratio with binding buffer, following the manufacturer’s instructions, and IgG-depleted sera were stored until needed for the assays. Total IgE and specific IgE against aGal conjugated to MSA or HSA were measured by sandwich enzyme-linked immunosorbent assay (ELISA) using the ELISA MAX ™ kit for IgE detection (Biolegends, CA, USA) following the manufacturer’s instructions. Plates were coated with mouse IgE capture antibody (Biolegends, CA, USA) or aGal-HSA or MSA (for the detection of specific IgE against aGal) at 5 µg/ml in 100 µl/well in carbonate-bicarbonate buffer. Washing was conducted after each step with PBST (0.05% Tween) using an ELx50 Auto Strip Washer (Bio-Tek instruments, Inc. VT, USA). Blocking buffer containing 1% bovine serum albumin (BSA, Akron Biotech, FL, USA) in PBST was used for blocking. For specific IgE against aGal, mouse sera were added at a dilution ratio of 1:1 (25 µl of serum/well) in blocking buffer and at a ratio of 1:50 for total IgE (2 µl of serum/well). A mouse IgE standard (Biolegends, CA, USA) was used for IgE quantification. For color development, we incubated the plate with 3,3′,5,5′-tetramethylbenzidine (TMB) (1-Step™ Ultra TMB-ELISA, Fisher Scientific, USA) for ~15 minutes. The reaction was stopped with 1 M phosphoric acid, and 450 nm absorbance was measured using a Varioskan Lux microplate reader (Thermo Fisher Scientific, MA, USA) using the SkanIt™ 6.0 software.

Specific IgG against aGal-HSA or MSA was measured by ELISA using the same protocol as the procedure described above. One difference was the secondary antibody step using goat-anti-mouse IgG-HRP (1:5000) overnight at room temperature.

### Chemicals and Other Compounds Used for Mice Immunizations and ELISA Assays

For immunizations we used the Freund’s incomplete adjuvant (Sigma Aldrich, MO, USA) in a 1:1 ratio of saline solution containing 25 µg of human serum albumin alone (Celprogen Inc. CA, USA) or conjugated to aGal containing a 14 atom spacer and an average of 25 aGal molecules per protein molecule (Dextra Laboratories UK) for mice immunizations. For the quantification of immunoglobulins against the aGal epitope we used aGal conjugated to HSA or MSA containing a 3 atom spacer and 33 aGal molecules per protein molecule (mouse serum albumin) (Dextra Laboratories, UK).

### Statistical Analysis

For statistical comparisons between treatment groups, a Kruskal–Wallis ANOVA test (one-way ANOVA nonparametric test) was conducted with a significance level of 0.05 (*p*<0.05). For comparisons between pre- and posttreatments, a paired sample Wilcoxon signed rank test with a significance level of 0.05 (*p*<0.05) was used. In addition to the ANOVA test, we tested the frequencies of the different individuals at the 95% normal distribution of the “sham” group. Chi-square tests were performed for the frequencies of the different individuals from the responses in the sham group in 2 x 2 contigency tables. Data analyses were conducted in OriginPro 2020b (9.7.5.184), GraphPad Prism version 9.2.0 for Windows (GraphPad Software, San Diego, CA, USA) and R version 4.0.0 -(R Foundation for statistical computing, Vienna, Austria).

## Results

### High Levels of aGal Epitopes in the Salivary Glands of Partially Fed Male *A. americanum*

**Figure 2:**
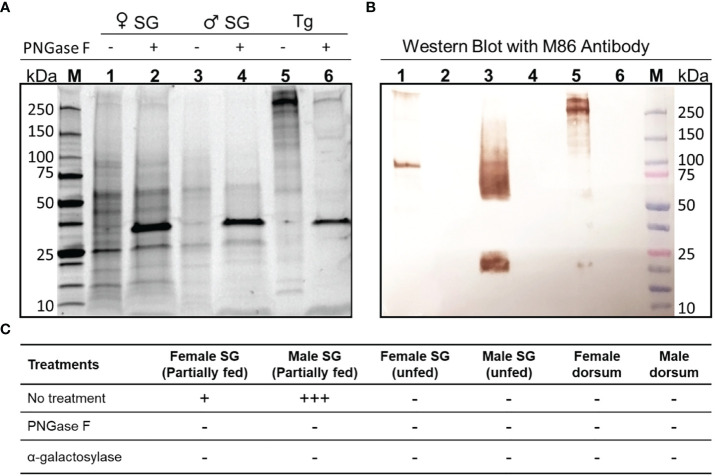
Western blot for aGal immunoreactivity in the salivary glands of female and male *A. americanum*. **(A)** SDS-polyacrylamide gel for the female and male salivary gland (SG) proteins (1.5 pairs per lane) and bovine thyroglobulin (Tg) as the positive control. The samples were treated with PNGase F (shown by ~35kDa bands in samples 2, 4, and 6). **(B)** Western blotting with mouse monoclonal anti- aGal IgM is shown. **(C)** Summary of repeated experiments with different types of samples (column headings) with different enzymatic deglycosylations. The ‘+’ indicates the arbitrary strengths of aGal reactivity, while the ‘-’ is for lack of the reactivity.

Western blots for salivary gland proteins from partially fed males and females showed high levels of reactivity to the M86 aGal monoclonal antibody, whereas the salivary glands of unfed ticks lacked reactivity (**Figure 2A**). Partially fed females revealed two anti-aGal reactive bands of ~100 kDa and very high molecular weight (Lane 1). The salivary glands of 4-day-fed male ticks contained high levels of aGal epitopes (**Figure 2B**) with a ~25 kDa band and a smear in the range of 50-200 kDa (Lane 3 in **Figure 2B**). The band patterns were consistent in five independent trials with three different tick batches using slightly modified protocols to avoid possible artifacts, such as protein degradation or overloading. The salivary glands of unfed ticks and the dorsum of the fed ticks did not show aGal epitopes (**Figure 2C**). The immunoreactivities of the partially fed tick salivary glands were abolished with PNGase F for deglycosylation of N-glycan (Lanes 2, 4, and 6 in **Figure 2B**) and with alpha galactosylase (deglycosylation of terminal aGal, **Figure 2C**). The reactivity in bovine thyroglobulin, the positive control containing ~4 aGal per molecule (60 pmoles of aGal in 10 μg thyroglobulin), indicated that the tick salivary glands contained high levels of aGal epitope, >60 pmoles per tick.

### Experiment 1: Alpha-Gal Sensitization in Mice by *A. americanum* Tick Bites

We present the pooled data for both males and females in the same treatment because the initial statistics for comparing males and females found no significant differences between sexes. We also show the data for various immunoglobulin levels in the posttreatments because we observed that all pretreatments were equally at the basal level (**Supplementary Figure S2A**) in the comparisons of total IgE and specific IgG against aGal-MSA and aGal-HSA (**Supplementary Figures S3A–C**). No signs of illness were observed in the mice, either after inoculation or tick infestation throughout our experiments.

#### *A. americanum* Tick Bites Increase the Levels of Total IgE and Specific IgE Against aGal

**Figure 3:**
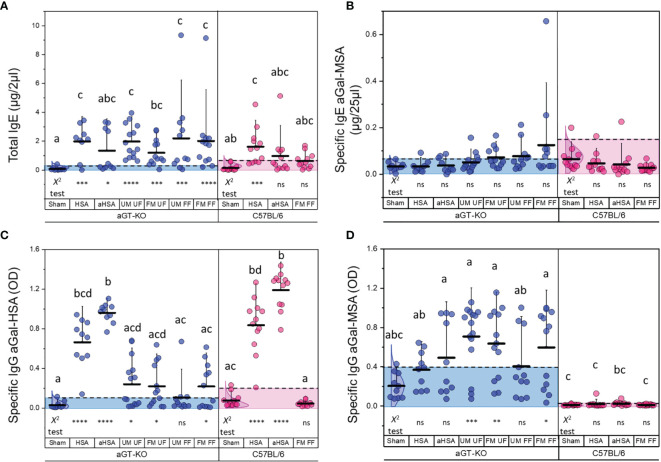
Total and specific IgE to aGal and specific IgG levels to aGal-HSA and aGal-MSA. **(A)** Total IgE levels of different treatment groups (in µg/2 µl of serum) in aGT-KO mice (blue) and C57BL/6 mice (magenta). **(B)** Specific IgE to aGal-MSA (in µg/25 µl of serum). **(C)** Specific IgG to aGal-HSA (OD value) and **(D)** Specific IgG to aGal-MSA (OD value). Note that the positive control for aGal is the immunization by aHSA when specific immunoglobulins against aGal-MSA were measured. Axis labels; HSA, human serum albumin; aHSA, aGal conjugated to HSA; UM, unfed male tick; UF, unfed female tick; FM, fed male tick; FF, fed female tick. The alphabets show the significant differences at *p* = 0.05 in Kruskal-Wallis ANOVA test. Lack of alphabets represents no statistical difference seen. Error bars represent the mean (lower horizontal bar) and standard deviation (upper vertical bars). Dotted lines and the shaded area show the cutoff for difference at 95% normal distribution of the control (sham control) for determining frequencies of differences, which was used for Chi-square test, and the statistics below the graph represent the Chi-square test results for significance indicated by *; *< 0.05, **< 0.01, ***< 0.001 and ns = not significant.

We measured the levels of total IgE among all treatment groups in aGT-KO mice and C57BL/6 wild-type (control) mice after treatment. All treatments in the aGT-KO mice showed higher levels of total IgE in comparison to the treatments in C57BL/6 control mice (**Figure 3A**). The HSA immunization, unfed male and female (UM, UF), and both fed female treatments (UM, FF, FM, FF) showed statistically significant differences when compared to the “sham” group in the ANOVA test (*p >*0.05) (**Table S1**). The chi-square test after the 95% distribution cutoff, providing the statistics for frequencies of individuals who were different from the responses of the sham control, showed significant differences across all treatments (**Table S2**), with a tendency of higher total IgE levels in the treatments with partially fed female (FF) ticks, with few individuals presenting a hyperreaction (**Figure 3A**). Highly significant differences in total IgE levels were observed between the “sham” and all tick feeding treatments.

Specific IgE levels against aGal-MSA, which is considered to be involved with the major contribution of specific IgE toward aGal, but not MSA, were significantly different in the tick feeding treatments that included any combinations of partially fed ticks when compared to the “sham” group in the chi-square test for frequencies of individuals with high levels of specific IgE. In the treatments of control mice, there were no significant differences in either the ANOVA or Chi square test for specific IgE against aGal-MSA (**Figure 3B**). In contrast to specific IgE against aGal-MSA, the levels of specific IgE against aGal-HSA were low in all treatments with the exception of a few individuals showing highly elevated specific IgE against aGal-HSA in the mice treated with aGal-HSA but not in the mice injected with HSA (**Supplementary Figure S1**).

#### Specific IgG Against aGal-HSA and to aGal-MSA

Specific IgG levels to aGal-HSA in the aGT-KO mice were significantly increased in both HSA and aHSA immunization (**Figure 3C**). Tick treatments resulted in significant levels of responses in the number of individual mice in all combinatory treatments for specific IgG against aGal-HSA, which is likely contributed by specific IgG against aGal, but not to HSA. C57BL/6 mice showed the highest levels of IgG against aGal-HSA under aGal-HSA treatment, whereas these mice showed no responses to tick feeding in terms of their specific IgG levels against aGal-HSA. The levels of specific IgG against aGal-MSA in the aGT-KO mice were generally high in the tick feeding groups (**Figure 3D**), while those in C57BL/6 mice were very low (**Figure 3D**), which suggests that specific IgG against aGal- MSA in the knockout mice is primarily against the aGal epitope.

### Experiment 2: Three Different Ixodid Tick Species in aGT-KO Mice

**Figure 4:**
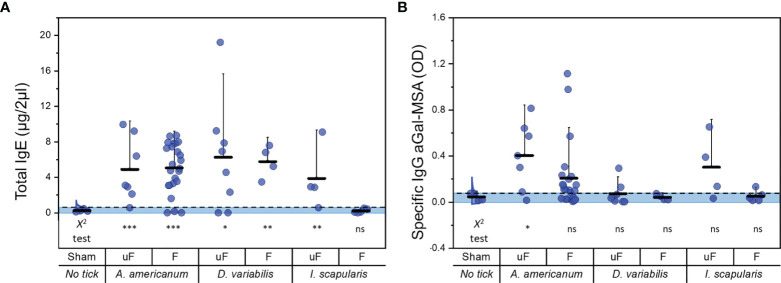
Comparisons of total IgE and specific IgG to aGal-MSA before and after feeding with different ixodid tick species. **(A)** total IgE (µg/2µl of serum) after tick infestation and **(B)** specific IgG to aGal-MSA (OD in 2µl of serum) after tick feeding. uF; unfed, F; partially fed (on bovine blood before infestation into mice), Aa; *A. americanum*, Dv; *D*. *variabilis*, Is; *I*. *scapularis*. Kruskal-Wallis ANOVA was conducted and the lack of alphabets represents no significant differences seen with this test. Dotted lines show the cutoff for Chi square test at 0.05. Shaded areas represent the data points within the 95% distribution cutoff. The statistics below the graph represent the Chi square test (*< 0.05, **< 0.01, ***< 0.001 and ns = not significant). Error bars represent the mean (lower horizontal bar) and standard deviation (upper horizontal bar).

We measured total IgE levels before and after treatment with different tick species. In this trial, our tick feeding time after tick attachment was longer than that in experimental segment A (8 ± 0.4 days). The Kruskal–Wallis ANOVA and the chi square test were performed as described above (**Tables S3** and **S4**). Tick feeding efficiently induced the elevation of total IgE levels in aGT-KO mice, except the case of partially fed *I. scapularis* (**Figure 4A**). The total IgE of the mice infested with unfed *I. scapularis* was also relatively low compared to the total IgE induced by feedings of other species of ticks.

At the levels of specific IgG against aGal-MSA, *A. americanum* treatments, both partially fed and unfed, induced (**Figure 4B**) high levels of the responses. Unfed *I. scapularis* also induced high levels of specific IgG in a number of individuals, while the levels were lowest in the feedings with *D. variabilis*.

#### Positive Correlation of Total IgE Levels With the Length of Tick Feeding

**Figure 5:**
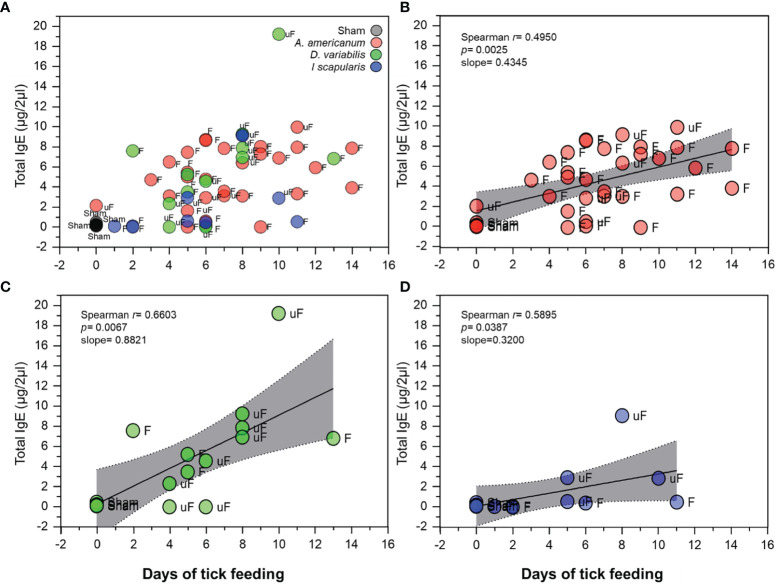
Correlation of total IgE levels with days of tick feeding. **(A)** Scatterplot showing total IgE levels (in µg/2µl of serum) in relation to days of tick feeding among the different treatments and tick species used. Gray; sham, Red; *A*. *americanum*, Green; *D*. *variabilis* and Blue; *I*. *scapularis*. Data labels; F; fed, uF; unfed. **(B)** Spearman’s rank correlation plot with linear regression, of *A*. *americanum* with days of tick feeding. **(C)** Spearman’s rank correlation plot with linear regression, of *D*. *variabilis* with days of feeding and **(D)** Spearman’s rank correlation plot with linear regression, of *I*. *scapularis* with days of feeding. Plots show positive correlation and linear regression slope. The gray shaded area represents the 95% confidence intervals.

In our experiments, the lengths of tick feeding were determined by the approximate size of the female tick reaching the level of the rapid engorgement phase. Therefore, the lengths of tick feeding varied in each case depending on how fast the female ticks reached their size indicating rapid engorgement phase. We found that the days of tick feeding and the total IgE levels of the mice were correlated (**Figure 5A**). The positive correlation in the linear regression with the highest slope was found in *D. variabilis* regardless of being unfed or partially fed (**Figure 5B**).

## Discussion

Tick bites have been considered to be the causal factors of AGS (10, 12), which was initially supported by the occurrence of AGS in subjects who have experienced tick bites (13). Additional support recently provided was using aGT-KO mice that lack aGal (23) as a model for the study of AGS (24, 25). Repeated injections of crude extracts of whole bodies of ticks or of tick salivary glands (8, 24) have both been shown to elevate the levels of specific IgE against the aGal epitope in aGT-KO mice. In this study, we addressed the contribution of male and female tick bites as sensitizers in AGS and tested tick function as the “transmitters” of the aGal epitope in aGT-KO mice. Direct tick feeding in our study, compared to the injection of tick extract (24, 25), could lead to differences in the host immune response.

High levels of aGal epitopes were found in the male salivary glands of ticks fed on bovine blood. Multiple experiments, 5 independent trials, showed similar results of unusual smeared band patterns in the western blots. We conclude that the smeared immunoreactivity on the blot likely presents true heterogeneity in the tick salivary gland or with glycoproteins having highly variable glycosylation patterns. The aGal epitopes that were abolished by PNGase F and a-galactosylase indicate that the aGal epitope is an N-glycan. The smeared band pattern in the male salivary glands can be caused by variable lengths of branches in glycosaminoglycans. N-glycosylation of highly branched muicin-like embryoglycan is an example of such a case (26, 27). Keratan sulfate I is also known to be a glycosaminoglycan with variable chain lengths anchored on asparagine (28). Large glycosaminoglycans were missed in our previous study using glycomics targeting small N-glycosylated aGal glycans (16, 17). Although the molecular identity of the aGal epitope in the male salivary glands awaits to be uncovered, the amount of aGal epitope in the male salivary gland is extremely high: >60 pmoles per tick based on the western blot compared to the standard of bovine thyroglobulin (**Figure 1**). Whether the aGal protein is directly assimilated from the host is unknown, while tick saliva is known to be rich in host proteins (16, 17, 29, 30), including the host immunoglobulins (31). *A. americanum* males are known to feed multiple times, while females rely on a single blood feeding for full engorgement (18). Together with the host-specific high levels of aGal in the saliva of male *A. americanum*, we found that *A. americanum* male feeding may play an important role depending on the prior host in the case of multiple feedings with an intrastadial host switch.

Dynamic control of the salivary components in tick feeding has been known. The factors that have been known to affect differential salivary gene expression and contents are the host species, host immune status, tick feeding stage over a week of feeding, and the presence of host pathogens (32–36). Interestingly, high levels of the aGal epitope in males fed on bovine blood were not observed in the case of male ticks fed on mice (see **Supplementary Figure S4**), which suggests that the enriched aGal epitope in the male salivary gland is host specific. The changed host blood, bovine to mouse, in our experiment may have affected the results through changes in tick salivary contents.

Several recent reports have also shown successful elicitation of AGS in aGT-KO mice and other animal models by multiple injections of crude extract of tick salivary glands or of whole body (8, 24, 25, 37). Direct tick feeding in this study compared to immunization with crude extract of tick salivary glands likely resulted in significant differences in the host response. The location of tick feeding is intradermal compared to subcutaneous injections in most immunization protocols, which results in large differences in the immune response (38). Tick feeding likely involves long-term, slow, sequential infusion of salivary components for a week (29), while needle-mediated injections are performed in a multiple-pulse manner over a long duration (24). Most significantly, the tick salivary components change over the feeding duration, which likely depends on the host and host immune responses. Despite difficulties in the experimental approach in direct tick feeding on the host, our study demonstrated that tick feeding causes elevated total IgE, specific IgG and specific IgE toward aGal in aGT-KO mice with large individual variations but not in C57BL/6 controls.

We questioned whether partially fed male ticks with bovine blood carrying high levels of aGal epitopes in the salivary glands efficiently elevate specific IgE against aGal. In the comparison between partially fed male ticks and naïve ticks, we were unable to find higher levels of specific IgE against aGal in ticks preconditioned with bovine blood. However, feedings with both treatments efficiently elevated specific IgE against aGal in aGT-KO mice with large individual variations. It is possible that the high levels of aGal in the saliva of bovine blood-fed ticks could have rapidly declined in the course of feeding on aGT-KO mice, as discussed above, resulting in no differences in the host response between the partially fed vs. unfed naïve tick treatments. We observed a lack of or low levels of aGal epitopes in the salivary glands after the ticks were fed on mice, in contrast to the case of artificial membrane feeding on bovine blood. It is also noted that a previous study reported that males of *A. americanum* that were unfed and partially fed on sheep blood lacked aGal epitopes in the salivary glands (17).

Large individual variations in mouse immune responses within the same treatments could be attributed to the differences among individual mice, the differences among ticks, and the inevitably different experimental procedures for tick feedings (i.e., the length of feeding). The contributions of the differences among individual mice to the individual variations were shown by the HSA and aHSA injection responses (**Figure 3**), which generally present lower levels of variation compared to the tick feeding treatments. Therefore, major causes of the differences among individuals appear to be the differences among ticks and the varied experimental procedures for tick feedings in the experimental sets. Individual differences in the protein content of the salivary glands in *A. americanum* during feeding on the same host were previously shown (39). Although this study does not support the aGal “transmission” hypothesis with the current set of data, our observations and previous reports together, as discussed above for changes in salivary contents by various factors, lead the future directions of the study to address understanding the dynamics of aGal contents in tick saliva depending on different hosts and on the immune status of the hosts. A recent report has also shown the variations in aGal levels in the tick salivary glands after feeding on different hosts (11). In addition, host-specific tick salivary proteins would be further supported by the saliva containing host proteins (29, 30). Furthermore, the male specific salivary proteins [i.e. immunoglobulin binding protein (40)] may contribute to the variation in our experimental system where a pair of ticks attached on adjacent feeding sites of the mouse.

Different tick species induced different levels of total IgE and specific IgG toward aGal in aGT-KO mice. *A. americanum* moderately induced total IgE but was most effective for increased specific IgG toward aGal. *D. variabilis* was the tick that induced total IgE the most efficiently, but with low levels of elevation of specific IgG toward aGal (**Figure 4**). *I. scapularis* was effective in the induction of specific IgG against aGal in two individual mice. The levels of induced total IgE were correlated with the length of feeding duration to different degrees in different species (**Figure 5**). On the other hand, C57BL/6 mice infected with *A. americanum* had very low levels of total IgE elevation but no changes in specific IgG and specific IgE, which is consistent with other studies that showed low or absence of immune response to tick feedings in wild-type mice (41, 42). These data imply that aGal is a major player in the immune response against ticks in aGT-KO mice. Therefore, different strategies for bypassing the host immune system, the basophil-based immune system against tick feeding in the dermal layer, can be employed in different tick species (25, 41).

Immunoglobulins for aGal are known to be an important component as protective antigens against human pathogens (43–45). Many pathogens, including those vectored by arthropods, carry aGal (46–50). A number of tick-associated bacteria have been identified as aGal producer, hence, a role for these bacteria in the sensitization of AGS has been proposed (51, 52). Bacterial association of the AGS needs to be further studied to understand the intricate interactions among the arthropod-host-microbe. In addition, a glyco aGal-based vaccine is known to effectively protect against Chagas infection in mice (53). Anti-aGal antibodies confer protection against malaria in mice (54). Understanding the signaling pathways for triggering protective antigens instead of sensitizing the allergic response would provide ways to improve human health.

## Data Availability Statement

The original contributions presented in the study are included in the article/**Supplementary Material**, further inquiries can be directed to the corresponding author.

## Ethics Statement

The mice were housed and bred in the AAALAC-approved animal facility within the Division of Biology, Laboratory Animal Care Service (LACS) at Kansas State University. Under the direction of Dr. Sherry Fleming (director of LACS), all animal protocols were approved and compliant with the guidelines of the Institutional Animal Care and Use Committee (IACUC) (IACUC #4228 and IACUC#4261).

## Author Contributions

LPM-R: conducted experiments, sample collection, sample processing, analysis, writing of the original manuscript draft. GDB: conducted experiments, sample collection, sample processing, analysis, data collection. DK: conducted tick dissections, tick feeding and processing of Western Blot and data collection. SDF: assisted with *in vivo* study design and implementation. YP: study design, conducted experiments, sample collection, analysis, writing, and reviewing. All authors contributed to the article and approved the submitted version.

## Funding

This work was conducted with the support of the U.S. Department of Defense; DOD TBDRP, D01 W81XWH-18-1-0255, and the K-INBRE from NIH grant no. P20GM103418, who supported the mice care personnel.

## Conflict of Interest

The authors declare that the research was conducted in the absence of any commercial or financial relationships that could be construed as a potential conflict of interest.

## Publisher’s Note

All claims expressed in this article are solely those of the authors and do not necessarily represent those of their affiliated organizations, or those of the publisher, the editors and the reviewers. Any product that may be evaluated in this article, or claim that may be made by its manufacturer, is not guaranteed or endorsed by the publisher.
