# Supplementary material for: Tick Intrastadial Feeding and Its Role on IgE Production in the Murine Model of Alpha-gal Syndrome: The Tick “Transmission” Hypothesis
Source: Front Immunol. 2022 Mar 4;13:844262. doi: 10.3389/fimmu.2022.844262 (PMC8930817; doi:10.3389/fimmu.2022.844262)
Supplement: Supplementary file 1 [file DataSheet_1.pdf]

Supplementary Material

Study of red meat allergy using a mouse model with bites of the Lone star ticks, *Amblyomma americanum*

1 Supplementary material

Supplementary Table S1A. Kruskal-Wallis ANOVA test of Total and specific IgE to aGal in Experiment 1

Supplementary Table S1B. Kruskal-Wallis ANOVA test of specific IgG to aGal in Experiment 1

Supplementary Table S2. Chi square test results after 95% distribution cutoff between sham group and treatments in aGT-KO and C57BL/6 control mice of Experiment 1

Supplementary Table S3. Kruskal-Wallis ANOVA test of total and specific IgE and IgG to aGal in Experiment 2.

Supplementary Table S4. Chi square test results after 95% distribution cutoff between sham group and treatments in aGT-KO and C57BL/6 control mice of Experiment 2

Supplementary Figure S1. Specific IgE to aGal-HSA.

Supplementary Figure S2. Comparisons of total IgE and specific IgG to aGal-MSA and aGal-MSA before and after feeding with *A. americanum*

Supplementary Figure S3. Comparisons of total IgE and specific IgG to aGal-MSA and aGal-MSA before and after feeding with *A. americanum* in the C57BL/6 control mice

Supplementary Figure S4. Western blot for aGal immunoreactivity in the salivary glands of male and female *A. americanum*, partially fed in bovine blood followed by direct mice feeding.

Table S1A. Kruskal-Wallis ANOVA test of Total and specific IgE to aGal in Experiment 1

	Total IgE*				Specific IgE HAS*				Specific IgE-MSA*			
Paired Comparisons	Mean Rank Diff	Z	Prob	Sig	Mean Rank Diff	Z	Prob	Sig	Mean Rank Diff	Z	Prob	Sig
SHAM HSA	-70.333	-4.1269	0.002	1	-6.8	-0.4164	1	0	6	0.35416	1	0
SHAM aHSA	-49.6	-2.9901	0.153	0	-27.75	-1.6993	1	0	2.79444	0.16923	1	0
SHAM UM UF	-76	-4.9486	4.1E-	1	1.41667	0.09503	1	0	-14.163	-0.9224	1	0
SHAM FM UF	-50.393	-3.2813	0.056	0	16.8591	1.05666	1	0	-33.01	-2.0436	1	0
SHAM UM FF	-63.5	-3.9181	0.004	1	7.58636	0.47548	1	0	-31.51	-1.9507	1	0
SHAM FM FF	-65.25	-4.1084	0.002	1	-5.6333	-0.3603	1	0	-34.097	-2.1516	1	0
SHAM SHAM_BI6	-7.0833	-0.446	1	0	11.0333	0.70567	1	0	-26.681	-1.6836	1	0
SHAM HSA_BI6	-65	-4.0927	0.002	1	26.4046	1.65492	1	0	-5.7639	-0.3637	1	0
SHAM aHSA_BI6	-39.083	-2.4608	0.762	0	55.7	3.56244	0.02021	1	8.36111	0.5276	1	0
SHAM FM FF_BI6	-35.833	-2.2562	1	0	60.5333	3.87156	0.00595	1	12.7778	0.8063	1	0
HSA aHSA	20.7333	1.21654	1	0	-20.95	-1.2829	1	0	-3.2056	-0.1941	1	0
HSA UM UF	-5.6667	-0.3576	1	0	8.21667	0.55117	1	0	-20.163	-1.3131	1	0
HSA FM UF	19.9405	1.25826	1	0	23.6591	1.48285	1	0	-39.01	-2.415	0.86	0
HSA UM FF	6.83333	0.40987	1	0	14.3864	0.90168	1	0	-37.51	-2.3222	1	0
HSA FM FF	5.08333	0.31079	1	0	1.16667	0.07462	1	0	-40.097	-2.5302	0.62	0
HSA SHAM_BI6	63.25	3.86701	0.006	1	17.8333	1.14058	1	0	-32.681	-2.0622	1	0
HSA HSA_BI6	5.33333	0.32607	1	0	33.2046	2.08112	1	0	-11.764	-0.7423	1	0

**HSA aHSA_BI6**	31.25	1.91058	1	0	62.5	3.99735	0.00352	1	2.36111	0.14899	1	0
**HSA FM FF_BI6**	34.5	2.10928	1	0	67.3333	4.30648	9.1E-04	1	6.77778	0.42769	1	0
**aHSA UM UF**	-26.4	-1.719	1	0	29.1667	1.95648	1	0	-16.957	-1.1396	1	0
**aHSA FM UF**	-0.7929	-0.0516	1	0	44.6091	2.79591	0.28465	0	-35.805	-2.2802	1	0
**aHSA UM FF**	-13.9	-0.8577	1	0	35.3364	2.21473	1	0	-34.305	-2.1846	1	0
**aHSA FM FF**	-15.65	-0.9854	1	0	22.1167	1.41453	1	0	-36.892	-2.3974	0.90	0
**aHSA SHAM_BI6**	42.5167	2.67702	0.408	0	38.7833	2.48049	0.72162	0	-29.475	-1.9155	1	0
**aHSA HSA_BI6**	-15.4	-0.9696	1	0	54.1546	3.39417	0.03786	1	-8.5583	-0.5562	1	0
**aHSA aHSA_BI6**	10.5167	0.66217	1	0	83.45	5.33726	5.2E-06	1	5.56667	0.36175	1	0
**aHSA FM FF_BI6**	13.7667	0.8668	1	0	88.2833	5.64639	9.0E-07	1	9.98333	0.64877	1	0
**UM UF FM UF**	25.6071	1.82651	1	0	15.4424	1.06533	1	0	-18.847	-1.3016	1	0
**UM UF UM FF**	12.5	0.8364	1	0	6.1697	0.42563	1	0	-17.347	-1.198	1	0
**UM UF FM FF**	10.75	0.7367	1	0	-7.05	-0.4985	1	0	-19.935	-1.41	1	0
**UM UF**	68.9167	4.72286	1.28E	1	9.61667	0.67997	1	0	-12.518	-0.8854	1	0
**UM UF HSA_BI6**	11	0.75383	1	0	24.9879	1.72384	1	0	8.39881	0.59405	1	0
**UM UF aHSA_BI6**	36.9167	2.5299	0.627	0	54.2833	3.83825	0.00682	1	22.5238	1.59312	1	0
**UM UF FM FF_BI6**	40.1667	2.75262	0.325	0	59.1167	4.18001	0.0016	1	26.9405	1.90551	1	0
**FM UF UM FF**	-13.107	-0.877	1	0	-9.2727	-0.5955	1	0	1.5	0.09788	1	0
**FM UF FM FF**	-14.857	-1.0182	1	0	-22.492	-1.4756	1	0	-1.0871	-0.0725	1	0
**FM UF SHAM_BI6**	43.3095	2.968	0.164	0	-5.8258	-0.3822	1	0	6.32955	0.42192	1	0
**FM UF HSA_BI6**	-14.607	-1.001	1	0	9.54545	0.61304	1	0	27.2462	1.81622	1	0
**FM UF aHSA_BI6**	11.3095	0.77504	1	0	38.8409	2.54815	0.59563	0	41.3712	2.75779	0.32	0

**FM UF FM FF_Bl6**	14.5595	0.99776	1	0	43.6742	2.86524	0.22918	0	45.7879	3.0522	0.12	0
**UM FF FM FF**	-1.75	-0.113	1	0	-13.22	-0.8673	1	0	-2.5871	-0.1725	1	0
**UM FF SHAM_Bl6**	56.4167	3.6437	0.014	1	3.44697	0.22614	1	0	4.82955	0.32194	1	0
**UM FF HSA_Bl6**	-1.5	-0.0969	1	0	18.8182	1.20857	1	0	25.7462	1.71623	1	0
**UM FF aHSA_Bl6**	24.4167	1.57696	1	0	48.1136	3.15649	0.08783	0	39.8712	2.6578	0.43	0
**UM FF FM FF_Bl6**	27.6667	1.78687	1	0	52.947	3.47358	0.02825	1	44.2879	2.95221	0.17	0
**FM FF SHAM_Bl6**	58.1667	3.84116	0.006	1	16.6667	1.11799	1	0	7.41667	0.5055	1	0
**FM FF HSA_Bl6**	0.25	0.01651	1	0	32.0379	2.10184	1	0	28.3333	1.93113	1	0
**FM FF aHSA_Bl6**	26.1667	1.72797	1	0	61.3333	4.11419	0.00214	1	42.4583	2.89386	0.20	0
**FM FF FM FF_Bl6**	29.4167	1.94259	1	0	66.1667	4.43841	5.0E-04	1	46.875	3.19489	0.07	0
**SHAM_Bl6**	-57.917	-3.8247	0.007	1	15.3712	1.00843	1	0	20.9167	1.42563	1	0
**SHAM_Bl6**	-32	-2.1132	1	0	44.6667	2.99621	0.15035	0	35.0417	2.38836	0.93	0
**SHAM_Bl6 FM**	-28.75	-1.8986	1	0	49.5	3.32042	0.04943	1	39.4583	2.68939	0.39	0
**HSA_Bl6**	25.9167	1.71146	1	0	29.2955	1.92192	1	0	14.125	0.96273	1	0
**HSA_Bl6 FM**	29.1667	1.92608	1	0	34.1288	2.23901	1	0	18.5417	1.26376	1	0
**aHSA_Bl6 FM**	3.25	0.21462	1	0	4.83333	0.32422	1	0	4.41667	0.30103	1	0

*Shaded areas show the significantly different comparisons.

Table S1B. Kruskal-Wallis ANOVA test of specific IgG to aGal in Experiment 1

	Specific IgG-HSA				Specific IgG-MSA			
**Paired Comparisons**	**Mean Rank Diff**	**Z**	**Prob**	**Sig**	**Mean Rank Diff**	**Z**	**Prob**	**Sig**
**SHAM HSA**	-70.6	-4.223	0.00133	1	-13.5	-0.8075	1	0
**SHAM aHSA**	-89	-5.3236	5.60E-06	1	-18.7	-1.1185	1	0
**SHAM UM UF**	-36.167	-2.3698	0.97888	0	-31.967	-2.0946	1	0
**SHAM FM UF**	-34.223	-2.1765	1	0	-28.915	-1.8389	1	0
**SHAM UM FF**	-19.8	-1.2122	1	0	-13.391	-0.8198	1	0
**SHAM FM FF**	-26.717	-1.6691	1	0	-25.05	-1.565	1	0
**SHAM SHAM_Bl6**	-23.383	-1.4609	1	0	47.0333	2.9384	0.18145	0
**SHAM HSA_Bl6**	-81.133	-5.0688	2.20E-05	1	47.075	2.941	0.17993	0
**SHAM aHSA_Bl6**	-99.967	-6.2454	2.32E-08	1	34.575	2.16007	1	0
**SHAM FM FF_Bl6**	-16.967	-1.06	1	0	46.5333	2.90716	0.2006	0
**HSA aHSA**	-18.4	-1.1006	1	0	-5.2	-0.311	1	0
**HSA UM UF**	34.4333	2.25622	1	0	-18.467	-1.21	1	0
**HSA FM UF**	36.3769	2.31345	1	0	-15.415	-0.9804	1	0
**HSA UM FF**	50.8	3.11012	0.10286	0	0.10909	0.00668	1	0
**HSA FM FF**	43.8833	2.74161	0.33626	0	-11.55	-0.7216	1	0
**HSA SHAM_Bl6**	47.2167	2.94986	0.17485	0	60.5333	3.78181	0.00856	1
**HSA HSA_Bl6**	-10.533	-0.6581	1	0	60.575	3.78441	0.00847	1

**HSA aHSA_Bl6**	-29.367	-1.8347	1	0	48.075	3.00348	0.1468	0
**HSA FM FF_Bl6**	53.6333	3.35074	0.04433	1	60.0333	3.75057	0.0097	1
**aHSA UM UF**	52.8333	3.46187	0.0295	1	-13.267	-0.8693	1	0
**aHSA FM UF**	54.7769	3.48363	0.02721	1	-10.215	-0.6497	1	0
**aHSA UM FF**	69.2	4.23662	0.00125	1	5.30909	0.32504	1	0
**aHSA FM FF**	62.2833	3.89115	0.00549	1	-6.35	-0.3967	1	0
**aHSA SHAM_Bl6**	65.6167	4.0994	0.00228	1	65.7333	4.10668	0.00221	1
**aHSA HSA_Bl6**	7.86667	0.49147	1	0	65.775	4.10928	0.00218	1
**aHSA aHSA_Bl6**	-10.967	-0.6851	1	0	53.275	3.32835	0.04805	1
**aHSA FM FF_Bl6**	72.0333	4.50028	3.73E-04	1	65.2333	4.07544	0.00253	1
**UM UF FM UF**	1.94359	0.13721	1	0	3.05128	0.2154	1	0
**UM UF UM FF**	16.3667	1.10292	1	0	18.5758	1.25178	1	0
**UM UF FM FF**	9.45	0.6527	1	0	6.91667	0.47772	1	0
**UM UF SHAM_Bl6**	12.7833	0.88293	1	0	79	5.45641	2.7E-06	1
**UM UF HSA_Bl6**	-44.967	-3.1058	0.10437	0	79.0417	5.45929	2.6E-06	1
**UM UF aHSA_Bl6**	-63.8	-4.4066	5.78E-04	1	66.5417	4.59594	2.4E-04	1
**UM UF FM FF_Bl6**	19.2	1.32612	1	0	78.5	5.42188	3.2E-06	1
**FM UF UM FF**	14.4231	0.94178	1	0	15.5245	1.01369	1	0
**FM UF FM FF**	7.50641	0.50159	1	0	3.86538	0.25829	1	0
**FM UF SHAM_Bl6**	10.8397	0.72433	1	0	75.9487	5.07503	2.1E-05	1
**FM UF HSA_Bl6**	-46.91	-3.1346	0.09464	0	75.9904	5.07781	2.1E-05	1
**FM UF aHSA_Bl6**	-65.744	-4.3931	6.15E-04	1	63.4904	4.24254	0.00122	1

**FM UF FM FF_Bl6**	17.2564	1.15311	1	0	75.4487	5.04162	2.5E-05	1
**UM FF FM FF**	-6.9167	-0.4433	1	0	-11.659	-0.7472	1	0
**UM FF SHAM_Bl6**	-3.5833	-0.2296	1	0	60.4242	3.87222	0.00593	1
**UM FF HSA_Bl6**	-61.333	-3.9305	0.00466	1	60.4659	3.87489	0.00587	1
**UM FF aHSA_Bl6**	-80.167	-5.1374	1.53E-05	1	47.9659	3.07384	0.11623	0
**UM FF FM FF_Bl6**	2.83333	0.18157	1	0	59.9242	3.84018	0.00676	1
**FM FF SHAM_Bl6**	3.33333	0.21841	1	0	72.0833	4.7232	1.3E-04	1
**FM FF HSA_Bl6**	-54.417	-3.5656	0.01997	1	72.125	4.72593	1.3E-04	1
**FM FF aHSA_Bl6**	-73.25	-4.7997	8.74E-05	1	59.625	3.90688	0.00514	1
**FM FF FM FF_Bl6**	9.75	0.63886	1	0	71.5833	4.69044	1.5E-04	1
**SHAM_Bl6 HSA_Bl6**	-57.75	-3.784	0.00849	1	0.04167	0.00273	1	0
**SHAM_Bl6 aHSA_Bl6**	-76.583	-5.0181	2.87E-05	1	-12.458	-0.8163	1	0
**SHAM_Bl6 FM FF_Bl6**	6.41667	0.42045	1	0	-0.5	-0.0328	1	0
**HSA_Bl6 aHSA_Bl6**	-18.833	-1.234	1	0	-12.5	-0.8191	1	0
**HSA_Bl6 FM FF_Bl6**	64.1667	4.20448	0.00144	1	-0.5417	-0.0355	1	0
**aHSA_Bl6 FM FF_Bl6**	83	5.43852	2.95E-06	1	11.9583	0.78356	1	0

Shaded areas show the significantly different comparisons.

Table S2. Chi square test results after 95% distribution cutoff between sham group and treatments in aGT-KO and C57BL/6 control mice of Experiment 1

	Total IgE	Specific IgE-MSA	Specific IgE-HSA	Specific IgG-HSA	Specific IgG-MSA
**CUTOFF FOR 95% DISTRIBUTION IN AGT-KO MICE**	**0.32533 (n=10)**	**0.06663 (n=9)**	**0.15766 (n=10)**	**0.09766 (n=10)**	**0.38288 (n=10)**
**NUMBER OF INDIVIDUALS OUTSIDE THE DISTRIBUTION (SHAM)**	**1**	**0**	**1**	**1**	**1**
**CHI SQUARE TEST RESULTS; P VALUE (N/TOTAL N FOR EACH TREATMENT); SIGNIFICANCE AT >0.05**					
**SHAM X HSA**	0.0006 (8/9)	0.3035 (1/9)	0.3049 (0/10)	<0.0001 (10/10)	0.0887 (5/10)
**SHAM X aHSA**	0.0510 (5/10)	0.0734 (3/10)	0.0191 (6/10)	<0.0001 (10/10)	0.0510 (5/10)
**SHAM X UM UF**	<0.0001 (14/14)	0.1364 (3/14)	0.2113 (0/15)	0.0270 (7/15)	0.0006 (12/15)
**SHAM X FM UF**	<0.0001 (12/13)	0.0431 (4/11)	0.2825 (0/11)	0.0121 (8/13)	0.0045 (9/13)
**SHAM X UM FF**	0.0023 (9/12)	0.0195 (5/11)	0.2825(0/11)	0.1566 (3/11)	0.3141 (4/11)
**SHAM X FM FF**	0.0006 (10/12)	0.0121 (6/12)	0.8923(1/12)	0.0449 (6/12)	0.0189 (7/12)
**CUTOFF FOR 95% DISTRIBUTION IN C57BL/6 MICE**	0.62062 (n=12)	0.15516 (n=12)	0.08336 (n=12)	0.20521 (n=12)	0.02556 (n=12)
**NUMBER OF INDIVIDUALS OUTSIDE THE DISTRIBUTION (SHAM)**	0	1	2	1	1
**CHI SQUARE TEST RESULTS; P VALUE (N/TOTAL N FOR EACH TREATMENT); SIGNIFICANCE AT >0.05**					
**SHAM X HSA**	0.0005 (8/12)	>0.999 (1/12)	0.5379 (3/11)	<0.0001 (12/12)	0.5371 (1/12)

**SHAM X aHSA**	0.0120 (5/12)	>0.999 (1/12)	0.1396 (0/12)	<0.0001 (12/12)	0.1779 (3/12)
**SHAM X FM FF**	0.0120 (5/12)	0.3070 (0/12)	0.1396 (0/12)	0.1396 (0/12)	0.5371 (0/12)

Table S3. Kruskal-Wallis ANOVA test of total and specific IgE and IgG to aGal in Experiment 2.

	Total IgE				Specific IgE-MSA				Specific IgE HSA			
	Mean Rank Diff	Z	Prob	Sig	Mean Rank Diff	Z	Prob	Sig	Mean Rank Diff	Z	Prob	Sig
**SHAM UF_AA**	-11.9444	-1.19754	1	0	0.53571	0.05241	1	0	2.57143	0.25155	1	0
**SHAM F_AA**	-12.413	-1.3805	1	0	0.25	0.02838	1	0	-0.16667	-0.01892	1	0
**SHAM UF_DV**	-16.75	-1.64796	1	0	-9.625	-0.96372	1	0	12	1.2015	1	0
**SHAM F_DV**	-10.25	-0.87334	1	0	-3.5	-0.30349	1	0	6.25	0.54194	1	0
**SHAM UF_IS**	-23	-1.9597	1	0	-23.75	-2.05942	0.82853	0	-7.5	-0.65033	1	0
**SHAM F_IS**	0.9	0.08083	1	0	-1.15	-0.10511	1	0	18.2	1.66351	1	0
**UF_AA F_AA**	-0.4686	-0.07181	1	0	-0.28571	-0.04078	1	0	-2.7381	-0.39082	1	0
**UF_AA UF_DV**	-4.80556	-0.59584	1	0	-10.1607	-1.20376	1	0	9.42857	1.117	1	0
**UF_AA F_DV**	1.69444	0.16988	1	0	-4.03571	-0.39479	1	0	3.67857	0.35985	1	0
**UF_AA UF_IS**	-11.0556	-1.10843	1	0	-24.2857	-2.37575	0.36778	0	-10.0714	-0.98522	1	0
**UF_AA F_IS**	12.84444	1.38741	1	0	-1.68571	-0.17652	1	0	15.62857	1.63652	1	0
**F_AA UF_DV**	-4.33696	-0.63659	1	0	-9.875	-1.48313	1	0	12.16667	1.82729	1	0
**F_AA F_DV**	2.16304	0.24056	1	0	-3.75	-0.42575	1	0	6.41667	0.72849	1	0
**F_AA UF_IS**	-10.587	-1.17741	1	0	-24	-2.7248	0.13512	0	-7.33333	-0.83256	1	0
**F_AA F_IS**	13.31304	1.62552	1	0	-1.4	-0.17462	1	0	18.36667	2.29077	0.46151	0
**UF_DV F_DV**	6.5	0.63951	1	0	6.125	0.61328	1	0	-5.75	-0.57572	1	0
**UF_DV UF_IS**	-6.25	-0.61491	1	0	-14.125	-1.41429	1	0	-19.5	-1.95244	1	0
**UF_DV F_IS**	17.65	1.8653	1	0	8.475	0.91152	1	0	6.2	0.66682	1	0
**F_DV UF_IS**	-12.75	-1.08635	1	0	-20.25	-1.75593	1	0	-13.75	-1.19228	1	0
**F_DV F_IS**	11.15	1.00142	1	0	2.35	0.2148	1	0	11.95	1.09225	1	0
**UF_IS F_IS**	23.9	2.14653	0.66844	0	22.6	2.06571	0.81598	0	25.7	2.34902	0.39528	0

Table S4. Chi square test results after 95% distribution cutoff between sham group and treatments in aGT-KO and C57BL/6 control mice of Experiment 2

	Total IgE	Specific IgE-MSA	Specific IgG-MSA
**CUTOFF FOR 95% DISTRIBUTION IN AGT-KO MICE**	**0.55075 (n=4)**	**0.05105 (n=4)**	**0.07407 (n=4)**
**NUMBER OF INDIVIDUALS OUTSIDE THE DISTRIBUTION (SHAM)**	**1**	**0**	**1**
**SHAM X uFAa**	0.0009 (7/7)	0.6650 (3/8)	0.0440 (6/8)
**SHAM X FAa**	0.0002 (21/24)	0.3184 (2/24)	0.1308 (15/23)
**SHAM X uFDv**	0.0143 (6/8)	>0.9999 (2/8)	>0.9999 (2/8)
**SHAM X FDv**	0.0047 (4/4)	0.2850 (0/4)	0. 2850 (0/4)
**SHAM X uFIs**	0.0047 (4/4)	0.1573(3/4)	0.1573 (3/4)
**SHAM X FIs**	>0.9999 (0/5)	0.8577 (1/5)	0.8577 (1/5)

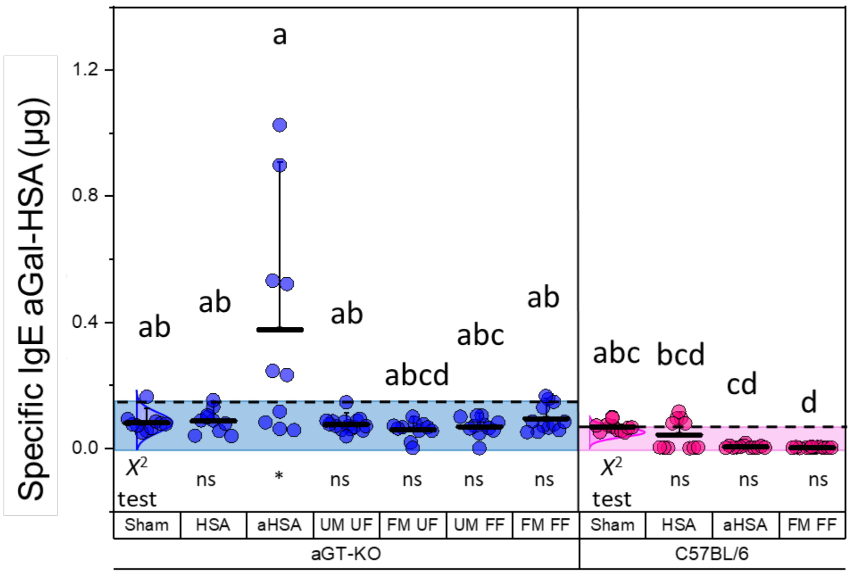

Figure S1. Specific IgE to α-Gal-HSA. Specific IgE to α-Gal-HSA [in micrograms (µg)] levels of different treatment groups in aGT-KO mice (blue) and C57BL/6 mice (magenta). Dotted lines show the cutoff for Chi square test at 0.157 for aGT-KO mice and 0.083 for C57BL/6 control mice. Shaded areas represent the data points within the 95% distribution cutoff. The alphabets show the significant differences at p=0.05 in Kruskal-Wallis ANOVA test and the statistics below the graph represent the Chi square test (ns=not significant and *<0.05). Error bars represent the mean (lower horizontal bar) and standard deviation (upper horizontal bar). Note that the positive control for α-Gal is the immunization by aHSA when specific immunoglobulins against α-Gal-MSA are measured. Axis labels; HSA, human serum albumin; aHSA, α-Gal conjugated to HSA; UM, unfed male tick; UF, unfed female tick; FM, fed male tick; FF, fed female tick.

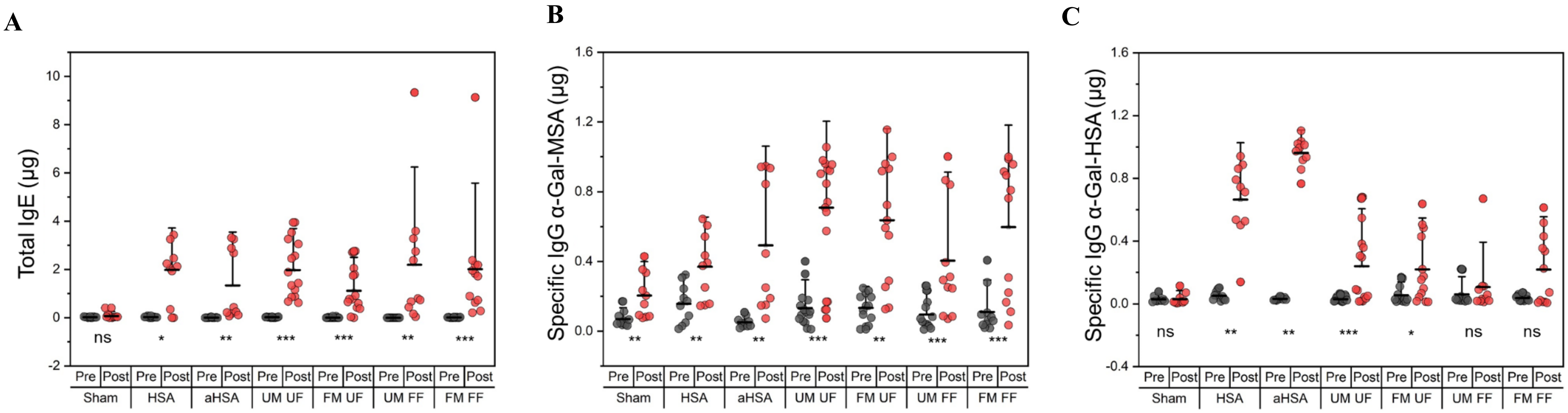

Figure S2. Comparisons of total IgE and specific IgG to α-Gal-MSA and α-Gal-MSA before and after feeding with *A. americanum*. A; total IgE before (pre) and after (post) treatment with partially and unfed male and female lone star ticks. B; specific IgG to α-Gal-MSA before and after tick feeding, and C; specific IgG to α-Gal-HSA before and after tick feeding, Axis labels; HSA, human serum albumin; aHSA, α-Gal conjugated to HSA; UM, unfed male tick; UF, unfed female tick; FM, fed male tick; FF, fed female tick. The statistics for difference between pre- and –post treatments were made by Wilcoxon signed rank test and shown by the star for the significance; *<0.05, **<0.01, ***<0.001 and ns= not significant.

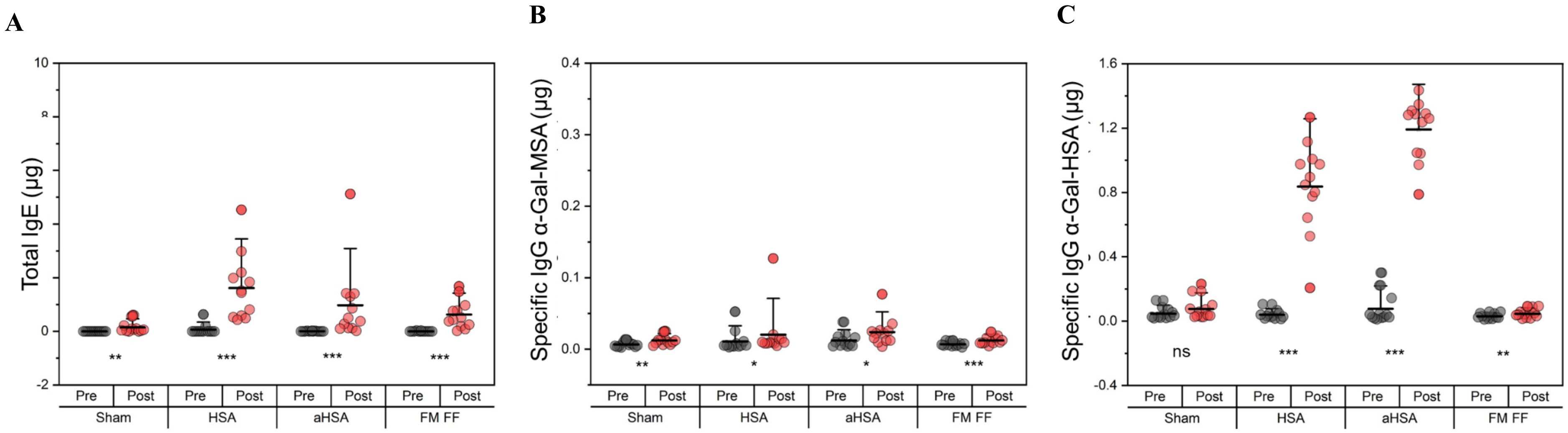

Figure S3. Comparisons of total IgE and specific IgG to α-Gal-MSA and α-Gal-MSA before and after feeding with *A. americanum* in the C57BL/6 control mice. A; total IgE before (pre) and after (post) treatment with partially and unfed male and female lone star ticks. B; specific IgG to α-Gal-MSA before and after tick feeding, and C; specific IgG to α-Gal-HSA before and after tick feeding, Axis labels; HSA, human serum albumin; aHSA, α-Gal conjugated to HSA; UM, unfed male tick; UF, unfed female tick; FM, fed male tick; FF, fed female tick. The statistics for difference between pre- and –post treatments were made by Wilcoxon signed rank test and shown by the star for the significance; *<0.05, **<0.01, ***<0.001 and ns= not significant.

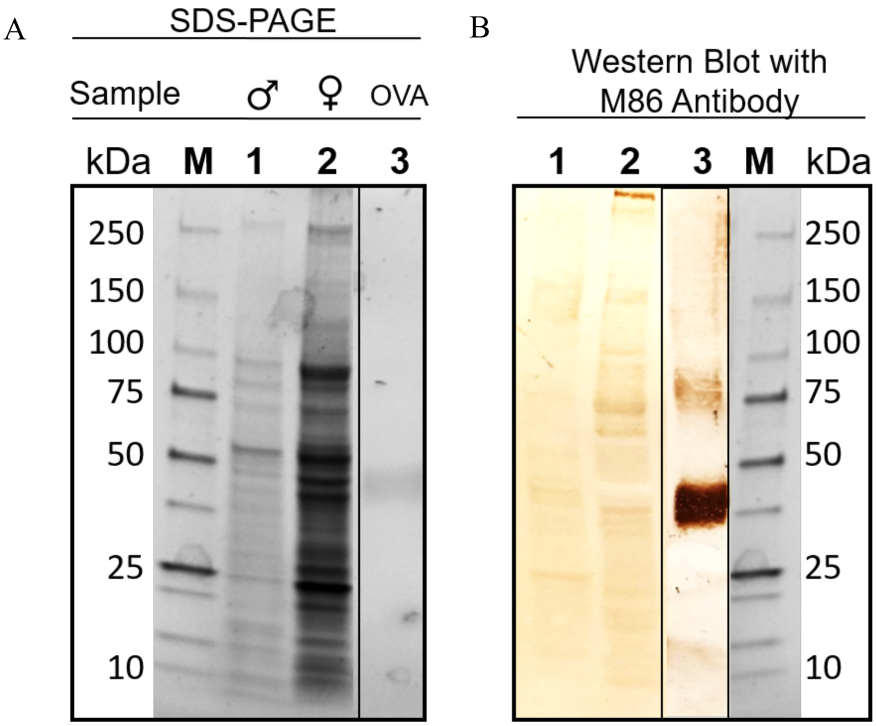

C

Well	Sample
1	SG of Partially fed *A.a* Male (Bovine Blood) followed by mice feeding (aGT-KO mouse)
2	SG of Partially fed *A.a* Female (Bovine Blood) followed by mice feeding (aGT-KO mouse)
3	Ovalbumin-alpha-Gal (~1 µg/lane)

Figure S4. Western blot for aGal immunoreactivity in the salivary glands of male and female *A. americanum*, partially fed in bovine blood followed by direct mice feeding. A; SDS-polyacrylamide gel for the male and female salivary gland (1/2 pair from single individual/lane), wells 1 and 2 respectively and ovalbumin conjugated to alpha-Gal (lane 3) as the positive control 1µg/lane (45 kDa containing ~10 molecules of aGal per protein molecule). B; Western blotting with mouse monoclonal anti- aGal IgM is shown. C; Summary of sample description used in Western Blot.
